# Minimally Invasive Tissue Sampling Findings in 12 Patients With Coronavirus Disease 2019

**DOI:** 10.1093/cid/ciab812

**Published:** 2021-12-15

**Authors:** Natalia Rakislova, Maria Teresa Rodrigo-Calvo, Lorena Marimon, Inmaculada Ribera-Cortada, Mamudo R Ismail, Carla Carrilho, Fabiola Fernandes, Melania Ferrando, Esther Sanfeliu, Paola Castillo, José Guerrero, José Ramírez-Ruz, Karmele Saez de Gordoa, Ricardo López Del Campo, Rosanna Bishop, Estrella Ortiz, Abel Muñoz-Beatove, Jordi Vila, Juan Carlos Hurtado, Mireia Navarro, Maria Maixenchs, Vima Delgado, Iban Aldecoa, Antonio Martinez-Pozo, Pedro Castro, Clara Menéndez, Quique Bassat, Miguel J Martinez, Jaume Ordi

**Affiliations:** 1 ISGlobal, Barcelona Institute for Global Health, Hospital Clínic-Universitat de Barcelona, Spain; 2 Department of Pathology, Hospital Clínic, Universitat de Barcelona, Spain; 3 Department of Pathology, Faculty of Medicine, Eduardo Mondlane University, Maputo, Mozambique; 4 Department of Pathology, Maputo Central Hospital, Maputo, Mozambique; 5 Department of Microbiology, Hospital Clínic, Universitat de Barcelona, Spain; 6 Centro de Investigação em Saúde de Manhiça, Maputo, Mozambique; 7 Catalan Institution for Research and Advanced Studies, Barcelona, Spain; 8 Pediatrics Department, Hospital Sant Joan de Déu, University of Barcelona, Spain; 9 Consorcio de Investigación Biomédica en Red de Epidemiología y Salud Pública, Madrid, Spain; 10 Neurological Tissue Bank of the Biobank, Hospital Clínic, Institut D’Investigacions Biomèdiques August Pi i Sunyer, University of Barcelona, Spain; 11 Medical Intensive Care Unit, Hospital Clínic, Institut D’Investigacions Biomèdiques August Pi i Sunyer, University of Barcelona, Spain

**Keywords:** minimally invasive tissue sampling, MITS, autopsy, COVID-19, SARS-CoV-2

## Abstract

**Background:**

Minimally invasive tissue sampling (MITS), a postmortem procedure that uses core needle biopsy samples and does not require opening the body, may be a valid alternative to complete autopsy (CA) in highly infectious diseases such as coronavirus disease-19 (COVID-19). This study aimed to (1) compare the performance of MITS and CA in a series of COVID-19 deaths and (2) evaluate the safety of the procedure.

**Methods:**

From October 2020 to February 2021, MITS was conducted in 12 adults who tested positive before death for COVID-19, in a standard, well-ventilated autopsy room, where personnel used reinforced personal protective equipment. In 9 cases, a CA was performed after MITS. A thorough histological evaluation was conducted, and the presence of severe acute respiratory syndrome coronavirus 2 (SARS-CoV-2) was evaluated by real-time reverse-transcription polymerase chain reaction (RT-PCR) and immunohistochemistry.

**Results:**

The diagnoses provided by MITS and CA matched almost perfectly. In 9 patients, COVID-19 was in the chain of events leading to death, being responsible for diffuse alveolar damage and mononuclear T-cell inflammatory response in the lungs. No specific COVID-19 features were identified. Three deaths were not related to COVID-19. All personnel involved in MITS repeatedly tested negative for COVID-19. SARS-CoV-2 was identified by RT-PCR and immunohistochemistry in the MITS samples, particularly in the lungs.

**Conclusions:**

MITS is useful for evaluating COVID-19–related deaths in settings where a CA is not feasible. The results of this simplified and safer technique are comparable to those of CA.

Coronavirus disease-19 (COVID-19) is caused by severe acute respiratory syndrome coronavirus 2 (SARS-CoV-2) [1]. In 2020, the rapid spread of COVID-19 to all continents led the World Health Organization to declare a global pandemic. In 2021, COVID-19 continues to take lives and disrupt health, economic, political, and social systems worldwide.

Clear evidence on the pathogenesis of COVID-19 is still lacking. COVID-19 damage primarily involves the lungs [2], although ongoing research focuses on exploring viral effects in other organs [3]. Autopsy-based studies were particularly scarce in the first half of 2020 [4], even though complete autopsy (CA) is the reference standard for evaluating emergent diseases [5, 6]. Logistical and biohazard challenges have been serious obstacles preventing the widespread use of CA. In addition to specific personal protective equipment (PPE), biosafety level 3 (BSL-3) or negative pressure rooms were usually listed as mandatory requirements to conduct autopsies after COVID-19 deaths in the first months of the pandemic [7, 8]. Later on, several guidelines, including those of the Centers for Disease Control and Prevention, recommended at least autopsy suites with negative air pressure, without requiring BSL-3 rooms [9]. These facilities are seldom available, even in high-income countries [10]. Moreover, although some guidelines do not require BSL-3 or negative pressure rooms, they instead recommend delaying the procedure ≥3 days [11], an unacceptable condition for most grieving families. Thus, these requirements are unfortunately difficult to meet, especially in low-resource settings [12]. Hence, there is a need for an alternative, less invasive, simpler, and safer approach to postmortem examinations in COVID-19 cases.

Minimally invasive tissue sampling (MITS) is a technique that consists of a series of needle-guided percutaneous punctures and core biopsies [13, 14]. A standardized MITS procedure [14], also known as a minimally invasive autopsy, has been validated [15, 16] and is currently being used as an alternative to CA for mortality surveillance in several low-income settings [17, 18]. MITS does not produce disfigurement of the body and, as a result, is more acceptable to relatives [19]. Remarkably, MITS has been safely conducted in other devastating contagious infectious diseases, such as yellow fever [20], tuberculosis [21], and Nipah virus [22], among others.

Herein, we report our preliminary results in a series of 12 MITS performed after death in adults with positive premortem polymerase chain reaction (PCR) COVID-19 test results. The current study aimed to (1) compare the diagnoses with MITS and CA performed on the same body and (2) evaluate the safety of MITS performed in a basic autopsy room following basic biohazard preventive measures (adequate use of PPE, with strict donning and doffing).

## METHODS

### Study Setting and Design

This observational postmortem study was conducted at the Pathology Department of the Hospital Clínic of Barcelona, Spain. We enrolled patients who died with a confirmed COVID-19 infection (positive reverse-transcription PCR [RT-PCR] assay result). MITS and CA were requested to clarify the cause of death or to evaluate the COVID-19–related damage in different organs. For all cases, consent for the 2 procedures (MITS and CA) was requested by the responsible clinician from the next of kin. Demographic and clinical data were retrospectively abstracted from the clinical records using a standardized questionnaire.

### MITS Procedure and Biosafety Measures

**Figure 1.**
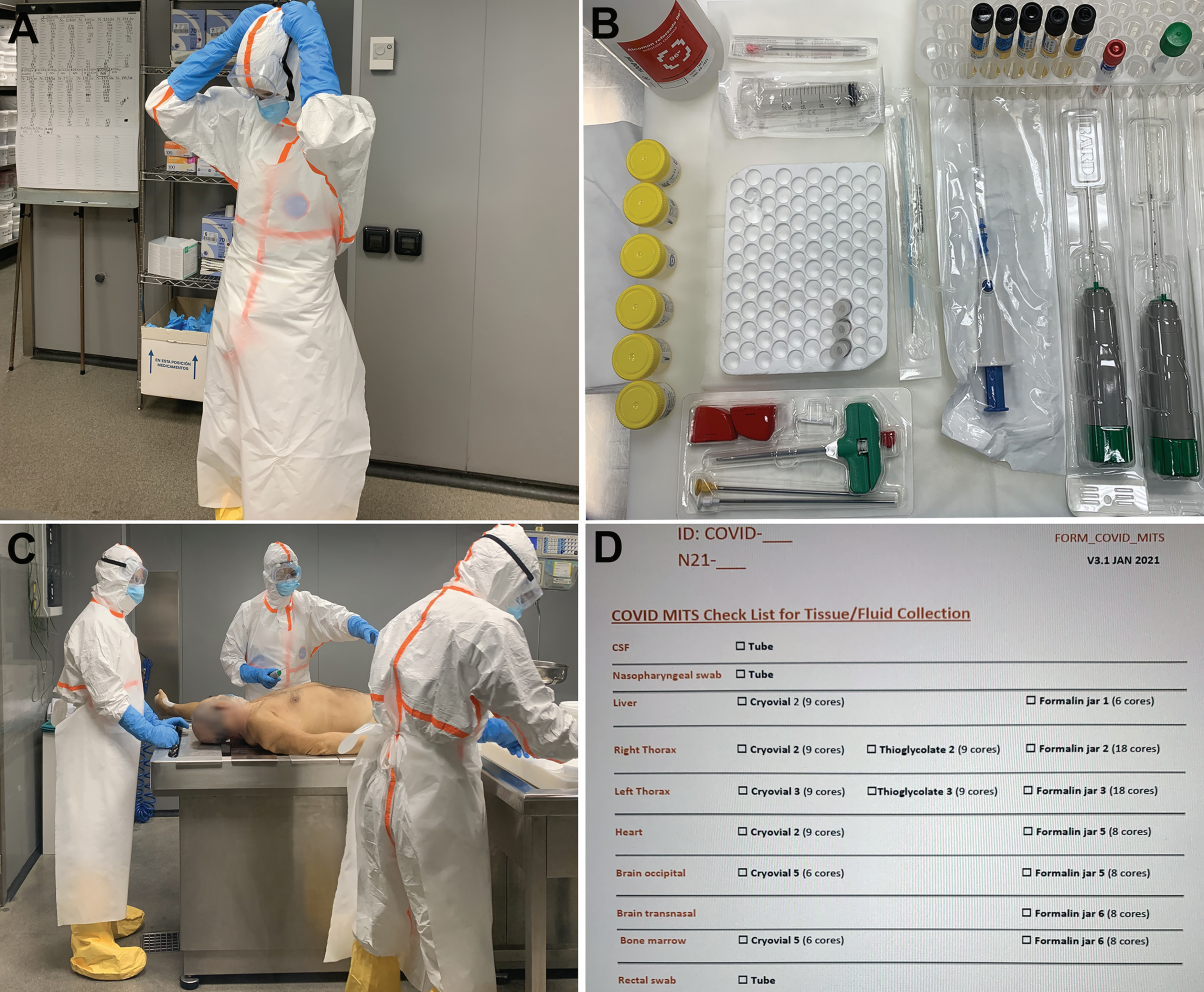
Minimally invasive tissue sampling (MITS) procedure in coronavirus disease 2019 (COVID-19) cases. *A,* Donning of personal protective equipment, including hooded waterproof coverall, apron, long-sleeved gloves, filter facepiece 3 mask under a surgical mask, and goggles. *B,* Contents of the coronavirus (COVID)–MITS kit box: 3 biopsy needles, 1 trephine, syringe with lumbar puncture needles, thioglycolate-filled tubes, formalin jars, cryovials, and containers for blood and cerebrospinal fluid, all labeled before the procedure. *C,* MITS sample collection process. One person performs the punctures (*center*), with the help of an assistant responsible of managing the tools and containers (*right*), and a second assistant (*left*), who helps in the movements of the body required for the procedure and with body transport before and after MITS. *D,* MITS checklist used for guiding and registering sample collection during the procedure.

The detailed sample collection and biosafety measures have been described elsewhere [12]. Briefly, we used a basic, well-ventilated autopsy room and reinforced PPE, which included filter facepiece 3 under a surgical mask, long-sleeved double gloves, scrub suit, waterproof hooded suit, apron, scrub hat, goggles, and waterproof shoe covers. Personnel with experience in MITS performed the procedure, either pathologists or pathology assistants; those >55 years old were specifically excluded from the team conducting the procedure. We used “COVID-MITS” kit boxes, which included 3 core biopsy needles, 1 syringe with lumbar puncture needle, 1 trephine, prelabeled containers and cryovials, swabs, and a checklist form for sample collection. Figure 1 shows the PPE, components of the COVID-MITS kit, and personnel conducting sample collection.

The procedure started with the collection of naso-oropharyngeal secretion swab samples, followed by extraction of cerebrospinal fluid from the cisterna magna with a lumbar puncture needle. Next, we conducted punctures of the liver, lungs, and heart using 14-gauge needles. The brain was sampled with a 16-gauge needle through transnasal and suboccipital punctures. Bone marrow samples were obtained using an 8-gauge trephine needle.

Before exiting the MITS room, all personnel removed their outer gloves and scrubbed the internal gloves with alcohol. The PPE doffing was performed as reported elsewhere [17]. After MITS and if CA was authorized, CA of the body was performed in a BSL-3 room. If CA was not indicated or consented to, the body was returned to the morgue. MITS histological samples were preserved in formaldehyde for 3 hours before they were paraffin embedded.

### CA Procedure

The CAs were conducted in a BSL-3 room by a different team of pathologists and pathology assistants, who were not involved in performing MITS. All thoracic and abdominal organs were eviscerated, dissected, and grossly examined. Samples were obtained from the main organs (both lungs, liver, kidneys, spleen, and bone marrow) and any visible lesions. Brain samples were not obtained as the cranial cavity was not opened. Samples were fixed in formaldehyde for 24 hours before the embedding process.

### SARS-CoV-2 Screening of the Personnel Involved in the Procedures

Two separate lists were created of the personnel participating in MITS and CA procedures, comprising 3 people in the MITS team (1 pathologist and 2 technicians) and 4 in CA team (2 pathologists and 2 technicians). All personnel on the MITS and CA lists underwent weekly SARS-CoV-2 rapid antigen testing (Roche Diagnostics Deutschland) until 3 weeks after the last MITS or CA procedure. Rapid antigen tests were conducted using the nasal swabs included in the test kits and following the instructions in the user manual. Thirty workdays before the study, all the participants underwent SARS-CoV-2 antibody serology testing. Circulating antibodies against SARS-CoV-2 were measured using Elecsys Anti-SARS-CoV-2 assay (Roche Diagnostics International), detecting antibodies against the nucleocapsid antigen.

### Histological Analysis

The slides with the tissue cores from MITS, as well as the sections from CA, were stained with hematoxylin-eosin. The samples obtained in each procedure (MITS and CA) were evaluated blindly to the other procedure by the same pathologists who performed the sampling (N. R. for MITS and I. A. for CA). The histological slides from MITS and CA for each case were evaluated together with the clinical and the microbiological data. Ancillary special and/or immunohistochemical (IHC) stains were requested if necessary.

### Microbiological Analyses: RT-PCR Assays and Cultures

The microbiological study included RT-PCR testing for COVID-19 in nasopharyngeal swab, cerebrospinal fluid, heart, liver, and lung samples obtained using MITS. Tissue samples were digested overnight in lysis buffer with proteinase K at 56ºC. Total nucleic acids were isolated using an automated platform (Magna Pure Compact; Roche). Then, the LightMix Modular SARS-CoV-2 E gene (TIB Molbiol) was amplified and detected in a LightCycler 480 II thermocycler (Roche). Duplicate biopsy samples were inoculated into thioglycolate broth–containing tubes and incubated at 37°C. If signs of bacterial or fungal growth were observed, the samples were subjected to Gram stain and subcultured into appropriate agar plates for identification.

### SARS-CoV-2 Detection by IHC

An anti-SARS-CoV-2 nucleocapsid protein antibody was used for IHC in MITS samples. A rabbit polyclonal nucleoprotein antibody (Sino Biological, 40143-T62; 1:1000) was used for IHC on formalin-fixed, paraffin-embedded MITS samples. Staining was applied to 4-μm-thick sections using an Autostainer Link 48 automated system (Dako) and the Envision system (Dako). The sections were counterstained with hematoxylin. Lung, liver, and heart tissues obtained in autopsies performed before the COVID-19 pandemic were used as negative controls. Placental tissue with PCR- and IHC-confirmed SARS-CoV-2 infection was used as a positive control. IHC evaluation was performed in 1 representative slide per organ and scored as negative (−) in the absence of any staining; positive (+) if 1–5 stained cells per slide were identified; positive (++) for 5–20 IHC-stained cells , and positive (+++) for >20 IHC-stained cells were recognized.

### Cause of Death Assignment

Each team included 1 pathologist, 1 microbiologist, and 1 physician with a background in infectious diseases. After discussing each case in online meetings, the corresponding team assigned the underlying, intermediate, and immediate causes of death for that case, as well as other conditions contributing to death, if any. The coding was performed following the *International Classification of Diseases, 11th Revision* (*ICD-11*). Each team was blind to the findings, diagnoses, and *ICD-11* codes of the other team.

Another investigator not involved in the MITS or CA teams (J. O.) compared the causes of death provided by both teams. Cases in which the MITS diagnoses (≥2 of 3 main causes of death) matched those of CA were deemed correctly classified.

### Ethics Statement

Ethical approval for MITS and CA procedures were obtained through the Institutional Review Board of the Hospital Clínic of Barcelona (HCB.2020.0577 and HCB.2020.0825). Verbal informed consent to perform the procedures was obtained from the next of kin. Written consent could not be obtained owing to visiting restrictions in the hospital and COVID-19 perimetral restrictions in Spain.

## RESULTS

### Study Cohort

Twelve patients, who died between 22 October 2020 and 12 February 2021, were included. Table 1 shows the patients’ demographic and clinical information, including symptoms, comorbid conditions, treatment, and type of postmortem examination consented to by the next of kin. Eight patients (66%) were male, and the median age was 76.3 years (range, 66–88 years). Ten patients (83%) had COVID-19 diagnosed with a PCR test of a naso-oropharyngeal swab sample during admission. Two patients (17%), who had been admitted for other diseases and were initially PCR negative for COVID-19, became COVID-19 positive during hospitalization.

**Table 1.** Clinical Characteristics of Enrolled Patients, Coronavirus Disease 2019 Treatment, and Type of Postmortem Examination Conducted

		Time to Death, d				Type of Autopsy
Patient/Age, y/Sex	Comorbid Conditions	From Symptom Onset	From Admission	Clinical Symptoms	COVID-19 Treatment	
1/78/M	Hypertension, diabetes mellitus type II, cerebrovascular disease	Unknown	3	Lethargy	Remdesivir, dexamethasone	MITS and CA
2/76/M	Hypertension	24	22	Fever, cough, hemoptysis, dyspnea, headache, anosmia	Remdesivir, dexamethasone	MITS and CA
3/85/F	Hypertension, chronic renal disease	3	1	Diarrhea, abdominal and thoracic pain	None^a^	MITS and CA
4/88/F	Diabetes mellitus type II, cerebrovascular disease, chronic renal disease	11	10	Jaundice, cough, dyspnea	Dexamethasone	MITS and CA
5/66/F	Chronic obstructive pulmonary disease, cor pulmonale, spondylitis, congestive heart failure	17	36^b^	Dyspnea, mucopurulent sputum	Remdesivir, tocilizumab	MITS
6/66/M	Diabetes mellitus type II, ischemic heart disease, kidney transplant, severe obesity	4	22^c^	Fever, dyspnea	Dexamethasone, convalescent plasma	MITS
7/84/M	Hypertension, dyslipidemia, atrial fibrillation, chronic renal disease, chronic lung disease, valvular heart disease	15	12	Fever, bronchorrhea, urinary and fecal incontinency	Remdesivir, dexamethasone	MITS
8/83/M	Diabetes mellitus type II, alcoholic liver disease, chronic renal disease, Barrett esophagus	8	3	General discomfort, asthenia, dyspnea	Convalescent plasma, dexamethasone	MITS and CA
9/75/M	Hypertension, obstructive sleep apnea	25	12	Somnolence, dyspnea	Dexamethasone	MITS and CA
10/66/M	Hypertension	20	12	Fever, aphonia	Dexamethasone, heparin	MITS and CA
11/68/M	Hypertension, chronic hydrocephaly	4	2	Fever, headache, somnolence, myoclonias	Remdesivir	MITS and CA
12/81/F	Hypertension, polymyalgia rheumatica, hypothyroidism	20	19	Fever, nausea, diarrhea, sweating, chest pain	Remdesivir, dexamethasone	MITS and CA

Abbreviations: CA, complete autopsy; COVID-19, coronavirus disease 2019; F, female; M, male; MITS, minimally invasive tissue sampling.

^a^Specific COVID-19 treatment was not applied because COVID-19 was diagnosed a few hours before the patient entered into cardiac arrest.

^b^Patient acquired COVID-19 while admitted to the hospital for treatment of chronic obstructive pulmonary disease.

^c^Patient acquired COVID-19 while admitted to the hospital for the study of potential disseminated cancer.

Patients had an average of 3 comorbid conditions, mainly arterial hypertension (8 cases; 66%), diabetes mellitus type II (4 cases; 33%), and chronic kidney disease (4 cases; 33%). Two patients had been treated with immunosuppressive drugs before acquiring COVID-19. The most common symptoms were fever and dyspnea (6 cases each; 50%).

The median time interval between the onset of symptoms and death was 8.4 days (range 4–17). Severe respiratory distress developed in 10 patients (83%), who required mechanical ventilation. All but 1 patient received ≥1 standard COVID-19 treatment (most frequently dexamethasone and/or remdesivir). One patient (patient 10) had COVID-19 with favorable evolution and was discharged after a negative test for COVID-19 on day 20 after symptom onset. After discharge, he experienced persistent fever with low back pain and was readmitted to the hospital, sudden death occurred during the second admission. Relatives provided consent for both MITS and CA in 9 patients and for MITS only in 3.

### SARS-CoV-2 Microbiological and IHC Results

Table 2 shows the detection of SARS-CoV-2 by RT-PCR and IHC in the postmortem samples obtained with MITS. In all cases, SARS-CoV-2 was detected in ≥1 sample by RT-PCR or IHC. The presence of virus was identified in ≥1 lung sample in all cases, with cycle threshold (Ct) values ranging from 20.9 to 37.4 and IHC scores between negative and positive (+++). SARS-CoV-2 was detected in both lungs in 9 of 12 cases (75%) by RT-PCR and by IHC in 11 of 12 (92%). All lung cultures were negative. SARS-CoV-2 RNA was consistently identified in all but 1 (92%) of the naso-oropharyngeal samples. Molecular detection of the virus by RT-PCR was less frequently in rectal swab (n = 5; 42%), liver (n = 4; 33%), heart (n = 3; 25%), and cerebrospinal fluid (n = 1; 8%) samples, with Ct values ranging from 25.6 to 37.6. IHC scores were negative in all liver, heart, and brain samples.

**Table 2.** Detection of Severe Acute Respiratory Syndrome Coronavirus 2 by Reverse-Transcription Polymerase Chain Reaction and Immunohistochemistry in Postmortem Samples Obtained With Minimally Invasive Tissue Sampling

	PCR and IHC Results by Sample Type^a^						
Patient	NP/OP Swab	Right Lung	Left Lung	Liver	Heart	CSF	Rectal Swab
1	18.2	25.6/+++	21.8/+++	−/−	34.2/−	−	31.7
2	32.6	30.5/++	31.2/++	−/−	−/−	−	34.9
3	30.5	−/−	37.4/−	−/−	−/−	−	30.2
4	17.6	31.0/+++	23.7/+++	33.0/−	31.7/−	NA	26.9
5	16.7	34.5/++	24.2/+++	NI/−	NA/−	−	−
6	16.3	20.9/+++	26.3/+++	36.6/−	NA/−	NA	−
7	28.0	28.1/+	26.0/+++	−/−	NA	−	−
8	34.2	25.0/+++	24.3/+++	−/−	−/−	36.2	−
9	17.0	30/+++	27.0/+++	−/−	−/−	NA	−
10	−	33.4/+++	−/++	−/−	−/−	−	−
11	21.8	25.0/+++	23.3/+++	31.3/−	−/−	NA	−
12	16.8	NI/+++	24.4/+++	31.6/−	25.64/−	−	34.5

Abbreviations: CSF, cerebrospinal fluid; IHC, immunohistochemistry; NA, no sample was available; NI, not informative (PCR-inhibited sample); NP/OP, naso-oropharyngeal; PCR, polymerase chain reaction.

^a^Numerical values represent cycle threshold (Ct) values for all cases with a positive result. The IHC result (score) was negative (−) in the absence of any stained cells, positive (+) with 1–5 stained cells identified per slide, positive (++) with 5–20 stained cells per slide, and positive (+++) with >20 stained cells per slide. Results for naso-oropharyngeal swab, rectal swab, and cerebrospinal fluid samples show only PCR results.

### Histological and IHC Findings of MITS and CA

**Figure 2.**
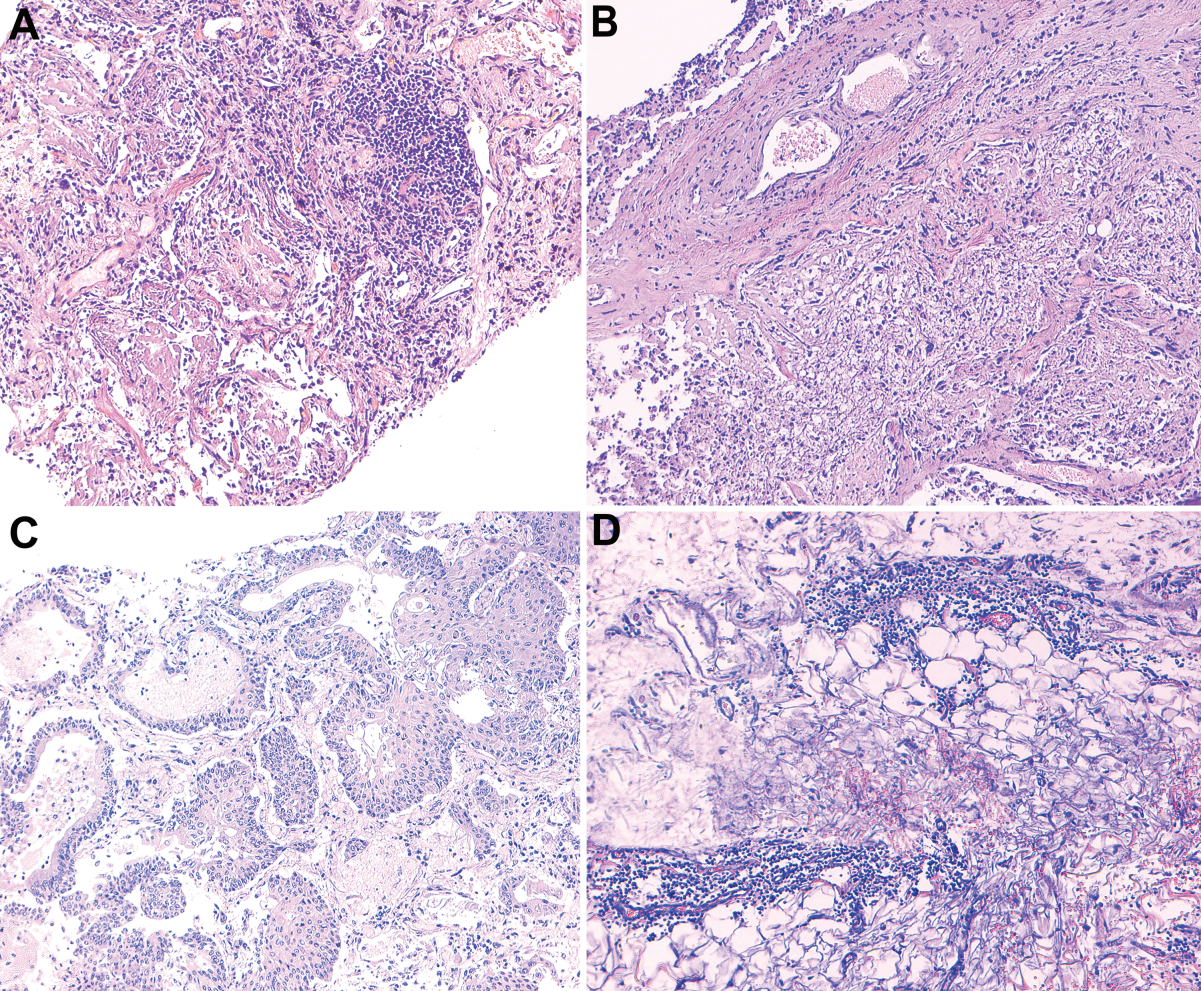
Spectrum of histological changes in the lungs identified by minimally invasive tissue sampling (MITS) and complete autopsy (CA) (*A–C*) and an example of an additional finding identified by CA but not by MITS (*D*). Diffuse alveolar damage (DAD) is a constant finding in the lungs damaged by coronavirus disease 2019 (COVID-19). *A–C,* Hyaline membranes (*A*), DAD in the fibroproliferative phase and recanalized microthrombi (*B*), and florid bronchiolar squamous metaplasia (*C*) are the common findings in the lungs of patients who died of COVID-19. *D,* Mononuclear lymphoid infiltrate in the pericardium in patient 12.

The main histological findings for MITS are shown in Table 3. Most of the relevant histological abnormalities seen with MITS were found within the lungs. Diffuse alveolar damage (DAD) at different stages, consistent with acute respiratory distress syndrome, was observed in 9 cases (75%). Edema, florid squamous bronchiolar metaplasia, multinucleated giant cells, and proliferation of pneumocytes type II and fibroblasts were commonly observed in these cases. Hyaline membranes were found in all cases with exudative DAD. Marked infiltration of CD163-positive macrophages was found in all cases with DAD. Figure 2A–Figure 2C shows the spectrum of histological lesions in the lungs with DAD.

**Table 3.** Histological Findings in Lungs and Other Organs With Minimally Invasive Tissue Sampling and Additional Findings From Complete Autopsy Only

	Findings at Both MITS and CA			
Patient	Lungs (Main Findings)	Lungs (Additional Findings)	Other Organs	Findings at CA Only
1	Necrotizing pneumonia	Aspiration, fungal hyphae	Liver steatosis	Kidney lithiasis, reactive lymphadenopathy
2	DAD (exudative or proliferative)	…	Hypercellular bone marrow	Tracheal ulcerations, hemorrhagic changes in pancreas, cardiomegaly
3	Focal interstitial infiltrates	…	Disrupted and infarcted myocardial tissue	Cardiac tamponade, severe atherosclerosis, coronary artery stenosis
4	DAD (exudative or proliferative)	Perivascular lymphoid inflammation; bronchiolar squamous metaplasia	Cholestatic hepatitis	Severe left kidney atrophy
5	DAD (proliferative)	Areas of liquefactive necrosis	Microthrombi	ND
6	DAD (exudative)	Fibrinous microthrombi	Extensive myocardial scarring	ND
7	DAD (exudative or proliferative)	Interstitial lymphoid infiltrates; bronchiolar squamous metaplasia		ND
8	DAD (exudative or proliferative)	Focal bronchopneumonia	Regenerative nodular hyperplasia in liver	Marantic endocarditis, submucosal lymphoplasmacytic infiltrate in trachea, arterial microthrombi in spleen, chronic perisplenitis
9	DAD (proliferative)	Extensive bronchopneumonia; Interstitial lymphoid infiltrates	Cholestasis, steatosis	Hepatosplenomegaly, cardiomegaly
10	Edema, congestion	…	Hypercellular bone marrow	Rupture of infected aortic aneurysm, hemoperitoneum, acute proliferative glomerulonephritis
11	DAD (exudative)	Interstitial lymphoid infiltrates; bone marrow fat embolism	Steatohepatitis; hypercellular bone marrow	Chronic pyelonephritis
12	DAD (exudative or proliferative)	Interstitial lymphoid infiltrates; corpora amylacea	Cholestasis	Mild pericarditis, pericardial microthrombi, chronic pyelonephritis

Abbreviations: CA, complete autopsy; DAD, diffuse alveolar damage; MITS, minimally invasive tissue sampling; ND, not done.

**Figure 3.**
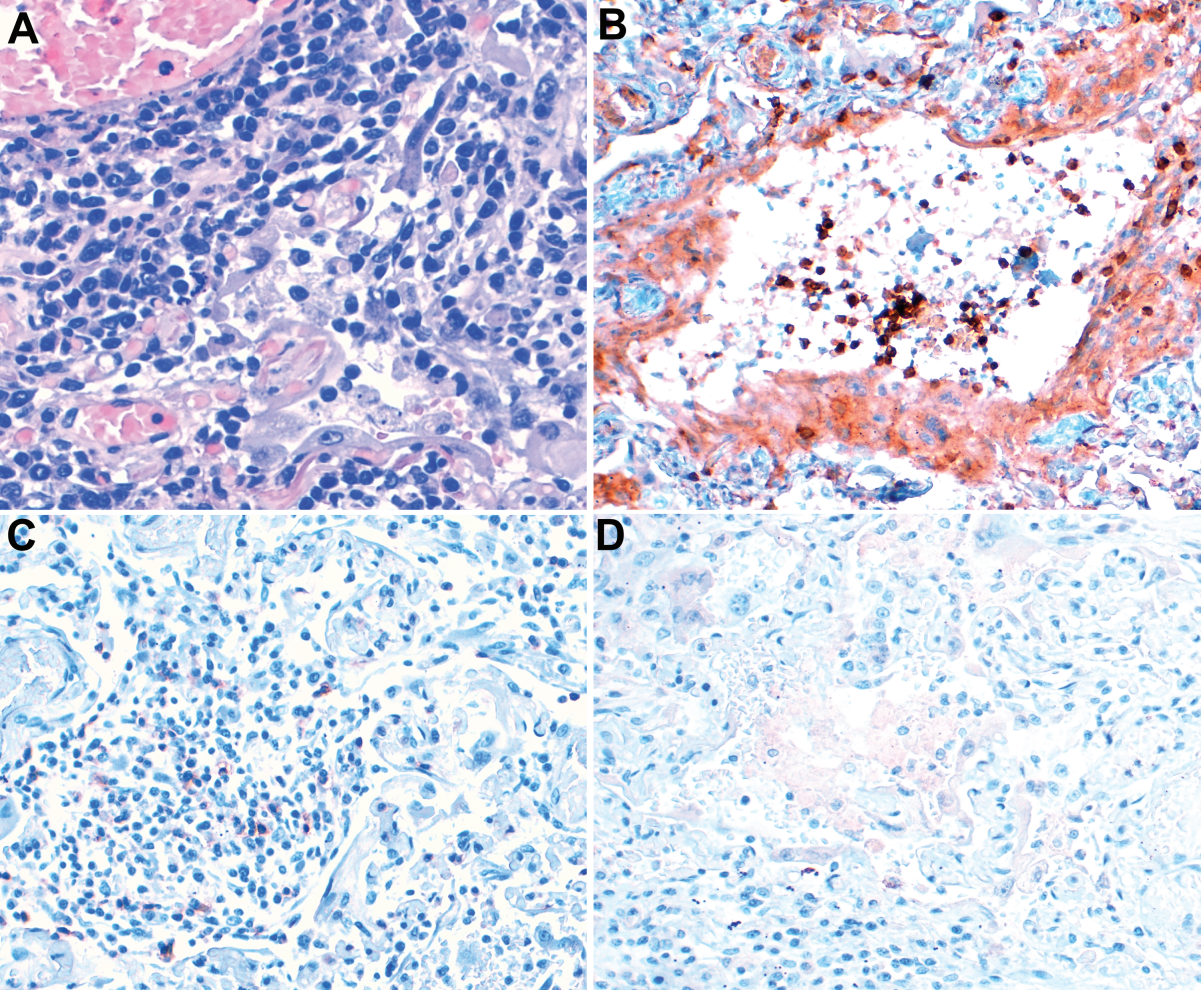
Immunohistochemical profile of the lymphoid inflammatory infiltrates identified in the lungs with diffuse alveolar damage associated with severe acute respiratory syndrome coronavirus 2 infection. *A*, indicates dense perivascular and interstitial lymphoid infiltrate. *B*, indicates expression of CD4. *C*, indicates scant CD8 cells. *D*, indicates absence of CD20-positive cells.

**Figure 4.**
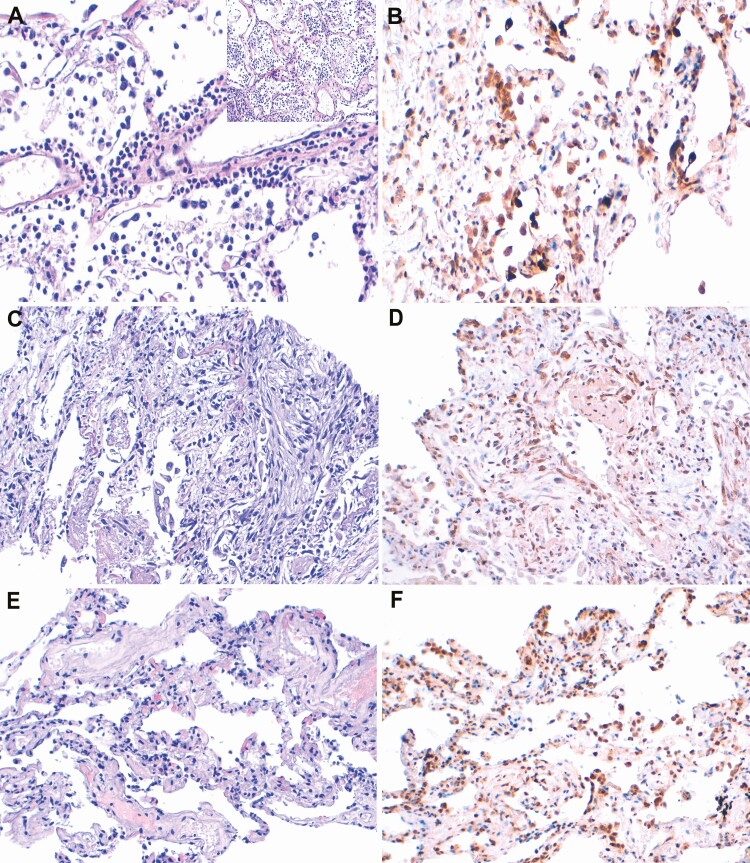
Immunohistochemistry (IHC) for severe acute respiratory syndrome coronavirus 2 (SARS-CoV-2) in the lung tissue of patients with different histological expressions. *A, B,* Findings in patient 1, with unknown time of symptoms onset. *A,* Florid bronchopneumonia with scant detached pneumocytes and perivascular inflammatory infiltrate. *B,* IHC shows abundant positive cells (+++; defined as >20 IHC-stained cells per slide) lining the alveoli and detached in the alveoli. *C, D,* Findings in patient 5, with SARS-CoV-2 symptoms lasting >2 weeks, include late-stage diffuse alveolar damage (*C*) and IHC positivity (++; defined as 5–20 IHC-stained cells per slide), predominantly in the lung interstitium (*D*). *E, F,* Findings in patient 10, who was discharged with a negative SARS-CoV-2 test result on day 20 after symptom onset and later died of aortic rupture, include lungs with edema and congestion (*E*) and diffuse (+++) IHC positivity in the cell lining of the alveoli (*F*).

Microthrombi were present in the lungs in 2 cases (1 with arterial microthrombi and 1 with fibrin plugs in the capillaries). No vasculitis or endothelitis was observed. Six cases showed lymphoid infiltrates in the lungs composed predominantly of CD4-positive T lymphocytes admixed with lower amounts of CD8-positive cells; CD68-CD163-positive macrophages were also abundant. CD20 lymphocytes and CD138 plasma cells were rare. Figures 3 and 4 show the IHC features of lymphoid inflammatory infiltrates and staining against SARS-CoV-2 nucleocapsid protein in the lungs, respectively. In 3 cases, there was no evidence of DAD or interstitial inflammation.

In case 1, the lungs showed prominent neutrophilic inflammation with necrosis and aspirated material, together with *Candida*-like hyphae, and focal presence of detached pneumocytes in the alveoli and perivascular mixed inflammatory infiltrates. Despite RT-PCR evidence of significant viral loads (low Ct values), IHC positivity for SARS-CoV-2 in the lungs, and the presence of viral RNA in other organs, the death was attributed to aspiration pneumonia, with COVID-19 considered a contributing condition. Case 3 showed hemorrhage and necrosis in the myocardial tissue, consistent with heart infarction and rupture. Only mild changes were observed in the lungs, which were correlated with low viral loads (high SARS-CoV-2 Ct values) and IHC negativity in the lungs. Finally, no relevant changes were identified by MITS in patient 10. In this patient’s body, only traces of SARS-CoV-2 RNA were detected in 1 lung but high IHC scores were observed in both lungs. The case was deemed nonconclusive by the MITS team.

The CAs confirmed all the histological findings observed with MITS. CA provided additional findings, including pericarditis (mixed CD20-positive and CD4-positive lymphoid infiltrate) with microthrombi in case 12. The pathological findings identified by CA but not MITS in the cases where both were performed are presented in Table 3.

### Cause of Death Attribution Based on MITS and CA Results

The cause of death chain of events and the factors contributing to death in each patient, as established by MITS and CA, are shown in Table 4. Among the 9 cases with both MITS and CA, 8 were correctly classified based on MITS, including all 6 patients with COVID-19–related DAD. A discordant diagnosis was observed in patient 10, who presented an aortic rupture that was not identified by MITS. In patient 3, MITS identified heart infarction and rupture but not a cardiac tamponade detected with CA.

**Table 4.** Diagnoses of Underlying, Intermediate, and Immediate Causes of Death and as Contributing Factors

	Diagnoses With MITS^a^				Diagnoses with CA^a^			
Patient	Underlying	Intermediate	Immediate	Contributing Factors	Underlying	Intermediate	Immediate	Contributing Factors
1	Aspiration pneumonia	Enterococcal septicemia	Septic shock	COVID-19; lung candidiasis	Aspiration pneumonia	Enterococcal septicemia	Septic shock	COVID-19; candidiasis
2	COVID-19	Pneumonia	ARDS	Hypertension	COVID-19	Pneumonia	ARDS	Hypertension
3	Acute myocardial infarction	Cardiac wall rupture	Cardiogenic shock	COVID-19	Acute myocardial infarction	Cardiac wall rupture	Cardiac tamponade	…
4	COVID-19	Pneumonia	ARDS	Cholestatic hepatitis	COVID-19	Pneumonia	ARDS	Cholestatic hepatitis; renal failure
5	COVID-19	Pneumonia	ARDS	Chronic obstructive lung disease	ND	ND	ND	NA
6	COVID-19	Pneumonia	ARDS	Immunosuppression after transplantation	ND	ND	ND	NA
7	COVID-19	Pneumonia	ARDS	Chronic lung disease	ND	ND	ND	NA
8	COVID-19	Pneumonia	ARDS	Liver disease	COVID-19	Pneumonia	ARDS	Liver disease; endocarditis
9	COVID-19	Pneumonia	ARDS	Bacterial pneumonia	COVID-19	Pneumonia	ARDS	Bacterial pneumonia
10	Non-conclusive	Nonconclusive	Nonconclusive	…	Aortic aneurysm	Aortic dissection	Hypovolemic shock	Acute glomerulonephritis
11	COVID-19	Pneumonia	ARDS	Steatohepatitis	COVID-19	Pneumonia	ARDS	Steatohepatitis
12	COVID-19	Pneumonia	ARDS	Drug-induced immunosuppression	COVID-19	Pneumonia	ARDS	Pericarditis

Abbreviations: ARDS: acute respiratory distress syndrome; CA, complete autopsy; COVID-19, coronavirus disease 2019; MITS, minimally invasive tissue sampling; NA, not applicable; ND, not done.

^a^All conditions were independently assigned by the death attribution teams for MITS and CA.

### SARS-CoV-2 Testing of Personnel Involved in Postmortem Procedures

All 6 study members involved in the postmortem examinations tested negative for SARS-CoV-2 antibodies before the onset of the study. All of them tested consistently negative for COVID-19 in the weekly antigen assays performed during the study period and 3 weeks after completion of the procedures.

## Discussion

The present study, which includes 12 patients, was conducted in a reference center with a heavy burden of COVID-19. All patients were older than 66 years and had comorbid conditions that favor an adverse clinical outcome of COVID-19 [23]. Although partial results of 6 of the cases presented in this series had been reported elsewhere [12], this study is one of the first to report results of MITS and CA performed on the same patients with COVID-19. Moreover, this is one of the first postmortem studies reporting the results of exhaustive SARS-CoV-2 testing performed on personnel who conducted the MITS procedures on bodies of patients with COVID-19 in a basic autopsy room, before vaccination.

The SARS-CoV-2–related damage was responsible for three-fourths of the deaths in our series. In keeping with data from the available CA [24–27] and MITS [28–34] studies performed on patients with COVID-19, viral lung damage, consisting of DAD in different stages, was the most consistent finding. In addition to DAD, macrophages as well as CD4 and CD8 T-cell lymphoid infiltrates were observed, whereas CD20 B cells (including CD138 plasma cells) were scarce or absent. These findings further confirm the evidence that the immune response in lung tissue is mainly regulated by T cells [26, 35]. In contrast with several MITS [28, 32] and CA [26, 36] series, the microthrombi were rarely noted in our patients (17%). Remarkably, SARS-CoV-2 has been identified in the lung samples obtained in the MITS procedures in all cases, in agreement with recent CA [37] and MITS [30] reports. Curiously, the lowest Ct values (<20) were usually observed in the naso-oropharyngeal swab samples, and not in the lungs. Another interesting point is the discordance between RT-PCR–positive results and negative IHC results in heart and liver. However, the presence of lung cores in heart and liver samples is common in MITS and might explain the viral RNA detection.

Importantly, MITS identified all the cases in which COVID-19 was directly causing death. These results confirm the high accuracy of MITS in diagnosing infectious diseases [15, 21, 38], including COVID-19 [12, 28]. Notably, during the first months of the outbreak, when CA was discouraged [4], MITS-based studies provided the first important insights into COVID-19 lung damage [39, 40].

COVID-19 was listed as a contributing factor in 2 patients who died of other conditions. The role of COVID-19 is questionable in patient 3, who died of heart rupture, as the PCR and IHC results were negative for SARS-CoV-2 in the heart tissue and no thrombotic features were identified. In this case, high Ct values (low viral load) were detected in the lungs and naso-oropharyngeal secretions, together with evidence of mild histological changes, probably indicating COVID-19 resolution with viral clearance. Consistently negative SARS-CoV-2 IHC staining results in all the tissues in this case are also in line with this hypothesis. Interestingly, another patient (patient 1) showed predominantly aspiration pneumonia, with scarce evidence of SARS-CoV-2–associated changes, although the virus was positive in both lungs with relatively low RT-PCR Ct values (high viral loads) together with high IHC scores (+++). These discrepant results might be related to the difficulties in identifying viral lesions in lungs with extensive aspiration pneumonia or, alternatively, to COVID-19 active viral replication with mild tissue damage.

Obviously, MITS also has some intrinsic limitations compared with CA, including its reduced capacity to identify gross anatomic abnormalities [13], as exemplified in case 10 of our series, which showed hypovolemic shock caused by a dissected aortic aneurism, not detected with MITS, although the procedure concluded that the death had not been caused by COVID-19. Other lesions identified by CA and not by MITS were associated conditions not involved in the chain of events leading to death.

Finally, the biosafety measures and the health safety and results of the personnel conducting postmortem examinations are of outmost importance in highly transmissible diseases, such as SARS-CoV-2 infection. Remarkably, patients with COVID-19 have shown to be infective up to 17 days after death [40]. Indeed, in terms of biosafety, MITS offers an advantage over CA: our experience indicates an extremely low probability of COVID-19 acquisition from the bodies when MITS is performed in a basic autopsy room without negative pressure [12]. However, reinforced PPE with appropriate respiratory and eye protection is strongly recommended and might be the key to safely performing these high-risk MITS [12].

Our study has some limitations. First, the number of cases is small. This reflects the logistical challenges that occurred in many institutions in conducting postmortem examinations during the COVID-19 outbreak. Second, the patients were ethnically homogenous and lived in a small geographic area (Barcelona metropolitan area).

In conclusion, our results highlight the value of MITS in identifying COVID-19–associated deaths and shows the high biosafety of the procedure even when performed in a basic autopsy room. The use of MITS could help expand the use of postmortem studies in COVID-19 patients and other highly transmissible infectious diseases, in both low- and high-resource settings. Our findings also confirm the absence of specific SARS-CoV-2 lesions.

## Notes

***Disclaimer.*** No funding bodies had any role in study design, data collection and analysis, decision to publish, or preparation of the manuscript.

***Financial support.*** This work was supported by the MITS Surveillance Alliance and by the Bill & Melinda Gates Foundation (grants OPP1067522, OPP1128001, and OPP1180554). ISGlobal is a member of the Centre de Recerca de Catalunya program, Generalitat de Catalunya. We received several grants by Bill&Melinda Gates foundation. This work was possible due to all these grants.

***Supplement sponsorship.*** This supplement is sponsored by MITS Surveillance Alliance Secretariat, led by RTI International, with funding from the Bill and Melinda Gates Foundation.

***Potential conflicts of interest.*** All authors: No reported conflicts. All authors have submitted the ICMJE Form for Disclosure of Potential Conflicts of Interest. Conflicts that the editors consider relevant to the content of the manuscript have been disclosed.
